# MCF-DTI: Multi-Scale Convolutional Local–Global Feature Fusion for Drug–Target Interaction Prediction

**DOI:** 10.3390/molecules30020274

**Published:** 2025-01-12

**Authors:** Jihong Wang, Ruijia He, Xiaodan Wang, Hongjian Li, Yulei Lu

**Affiliations:** 1 School of Computer, Guangdong University of Education, Guangzhou 510310, China; 2 School of Chemistry and Chemical Engineering, Guangdong Pharmaceutical University, Zhongshan 528458, China

**Keywords:** drug-target interactions, MSCNN, Transformer, BFIM, SFM

## Abstract

Predicting drug–target interactions (DTIs) is a crucial step in the development of new drugs and drug repurposing. In this paper, we propose a novel drug–target prediction model called MCF-DTI. The model utilizes the SMILES representation of drugs and the sequence features of targets, employing a multi-scale convolutional neural network (MSCNN) with parallel shared-weight modules to extract features from the drug side. For the target side, it combines MSCNN with Transformer modules to capture both local and global features effectively. The extracted features are then weighted and fused, enabling comprehensive feature representation to enhance the predictive power of the model. Experimental results on the Davis dataset demonstrate that MCF-DTI achieves an AUC of 0.9746 and an AUPR of 0.9542, outperforming other state-of-the-art models. Our case study demonstrates that our model effectively validated several known drug–target relationships in lung cancer and predicted the therapeutic potential of certain preclinical compounds in treating lung cancer. These findings contribute valuable insights for subsequent drug repurposing efforts and novel drug development.

## 1. Introduction

The identification of drug–target interactions (DTIs) plays a crucial role in the early stages of drug discovery and drug repurposing [1]. However, large-scale biological and chemical experiments in the laboratory for DTI identification are time-consuming and costly [2]. With the rapid development of data and computational technologies, researchers increasingly prefer computational approaches that can provide innovative methods to predict DTIs quickly and accurately. In recent years, from a model perspective, methods based on traditional machine learning [3,4,5], deep learning [6,7,8], and graph neural networks (GNNs) [9,10,11] have gradually become the dominant approaches for DTI prediction. These methods have significantly advanced the field of DTI research, making drug discovery, drug repurposing, and especially targeted drug development for cancer faster and more precise. The prediction of drug–target interactions can also be analyzed from the perspective of feature contributions of drugs and targets themselves. DTI identification can be categorized into docking-based methods [12], ligand-based methods [13], and chemogenomics-based methods [14,15]. Docking-based methods primarily rely on the three-dimensional (3D) structural data of drug molecules and target proteins to simulate binding positions, affinities, and energies, thereby predicting the likelihood of interactions [16]. However, when the 3D structure of a protein is unknown, the applicability of these methods is significantly limited [17]. Ligand-based methods rely on known ligand information to predict interactions between new ligands and specific targets by comparing the structures and activities of new molecules with those of known ligands. The most commonly used ligand-based prediction approach is the quantitative structure–activity relationship (QSAR) method [18]. Chemogenomics-based methods explore the specific interactions between small molecule drugs and target proteins to study the structure and function of the target proteins and related genes, thereby identifying new drug targets and lead compounds [19].

Feature engineering plays a crucial role in improving DTI prediction; however, it faces significant challenges due to the complexity of drug and target features as well as the high-dimensional nonlinear relationships between them. Designing an efficient model capable of automatically extracting features and deeply modeling the interaction between drugs and targets is therefore of great importance. CNNs, Transformers, and their variants have achieved remarkable success in various fields, making them promising approaches to explore for advancing DTI prediction.

Based on the above analysis, we aim to propose a DTI prediction model that is simple, effective, and broadly applicable without relying on complex, manually constructed features. We present MCF-DTI, a model that leverages the properties of drugs and target proteins, using multi-scale convolutional networks and Transformer modules to extract both local and global features from drug SMILES representations and target sequences. Additionally, the model incorporates shared weights, feature interaction, and selective feature extraction to enhance predictive power.

In this study, our contributions are as follows:

1. We propose a local–global feature fusion model that combines multi-scale convolution with Transformer architecture. Specifically, the model integrates MSCNN and Transformer in a parallel convolution manner to capture distinctive features of the target-side sequences. Subsequently, feature crossing and feature selection are applied to enhance the model’s predictive capability.
2. The proposed model does not require complex feature engineering of the original input data, resulting in a simple yet effective approach with strong applicability. Compared to several existing models, our model demonstrates superior predictive performance.
3. We validated our model using known lung cancer targets and predicted potential therapeutic drugs for several lung cancer targets. This work highlights new drug discoveries and the possibilities for drug repurposing in targeted lung cancer therapy.

## 2. Related Work

In recent years, many DTI prediction methods based on traditional machine learning, deep learning, and GNN have been proposed. André C. A. Nascimento et al. [20] introduced a multi-kernel machine learning method named KronRLS-MKL, which modeled drug–target interactions as a link prediction problem on a bipartite network. Experimental results demonstrated that this method outperformed 18 competing approaches across multiple datasets. Ming Wen et al. [21] developed a deep learning framework called DeepDTIs, which employed unsupervised pre-training to abstract representations from raw inputs, followed by constructing a classification model using known labeled interactions, achieving an accuracy of 91.58%. Zhen Tian et al. [22] proposed a model named CCL-ASPS, which combines Collaborative Contrastive Learning (CCL) and Adaptive Self-Paced Sampling (ASPS) strategies for DTI prediction, achieving high accuracy.

Among GNN-based methods, Pinglu Zhang et al. [23] proposed the Meta Graph Association-Aware Contrastive Learning (MGACL) model, which utilizes heterogeneous graph neural networks in combination with a contrastive learning framework for DTI prediction. Kanghao Shao et al. [24] treated DTI prediction as a link prediction problem and proposed DTI-HETA, an end-to-end model based on heterogeneous graph attention mechanisms. Additionally, Huang et al. [25] introduced DeepPurpose, a comprehensive and user-friendly deep learning library for DTI prediction.

From the perspective of feature construction, Qin Zhang et al. [26] utilized molecular docking to study the potential mechanisms of Si-Ni-San in the treatment of hepatocellular carcinoma (HCC), but the docking process is typically time-consuming and restricted by the availability of protein 3D structures. Yannan Li et al. used the QSAR method to investigate the inhibitory effects of flavonoids on CYP3A4, demonstrating the capability of feature prediction without relying on target 3D structures [27]. However, these methods face challenges such as unclear physical meaning of the regression equations and reduced model performance when the available information is insufficient.

Feature reconstruction methods based on deep learning have shown significant performance advantages. For example, Ingoo Lee et al. [28] constructed a CNN-based DTI prediction model to extract local patterns from protein sequences, outperforming other traditional descriptors. Qianwen Lu et al. [29] developed a Multi-Layer Graph Attention Neural Network (MLGANN), which combines Graph Convolutional Networks (GCN) with self-attention mechanisms for DTI prediction, achieving superior results. Huaihu Li et al. [30] proposed the MdDTI model, which utilized a drug molecular graph-based substructure decomposition strategy. Compared to SMILES-based approaches, MdDTI demonstrated higher accuracy (93.8%) and showed potential for drug repurposing. The ConformerDTI [31] model leverages CNN and Transformer to extract features from the SMILES representations of drugs and the amino acid sequences of proteins, enabling accurate DTI prediction.

By combining GNN and Transformer modules, the performance of DTI prediction models can be further enhanced. For example, Liu et al. [32] proposed the GraphDTI model, which utilizes graph neural networks to encode molecular graphs and employs self-attention mechanisms to strengthen feature interactions, achieving state-of-the-art performance across multiple datasets. Additionally, Chen et al. [33] adopted a Transformer-based architecture that integrates sequence features and molecular descriptors, significantly improving prediction sensitivity and specificity. Similarly, GraphormerDTI [34] incorporates graph Transformer techniques to represent drug molecules as molecular graphs while integrating global features from target sequences, effectively capturing the complex interactions between drugs and targets.

The advancements in CNN, Attention, GNN, and Transformer-based deep learning methods have demonstrated significant progress in DTI prediction. By leveraging deep learning techniques to reconstruct features for drugs and targets, researchers have effectively utilized existing data to build high-performance DTI prediction models. However, there remains considerable room for further development. In this study, we propose the MCF-DTI model, which employs multi-scale convolutional networks and Transformer modules to automatically learn local and global features from drug SMILES representations and target sequences. These features are then subjected to cross-feature interaction and selective fusion, showcasing the immense potential of deep learning for DTI prediction.

## 3. Results and Analysis

### 3.1. Ablation Experiment

To better analyze the influence of each module in the MCF-DTI model on the DTI prediction results, we conducted a series of ablation studies. These ablation experiments were designed to systematically investigate the contribution of different model components to the overall predictive performance.

MSCNN-MSCNN: This model applies a single MSCNN to both drug and target features simultaneously. The goal is to examine the effect of multi-scale feature extraction on both sides without additional processing.

MSCNN2-MSCNN2: In this model, the base features of both drug and target are processed through two MSCNNs. The features from these two stages are then concatenated in parallel to make the DTI prediction.

MSCNN-Transformer: This model uses a single MSCNN to extract features from the drug, while relying solely on a Transformer for feature extraction from the target protein. This design aims to specifically highlight the contribution of the Transformer in capturing global target features.

MSCNN2-MSCNN+Transformer (S): The model primarily utilizes both MSCNN and Transformer in parallel on the target side to effectively capture both local and global features, enhancing the model’s feature extraction capabilities.

The overall results of these ablation experiments are summarized in Table 1. These experiments help in understanding the role of individual modules in the MCF-DTI model and demonstrate the effectiveness of each component in improving the accuracy and robustness of DTI prediction.

From Table 1, it can be observed that due to the typically short nature of drug molecule SMILES strings, employing either a single MSCNN or parallel MSCNN for multi-scale local feature extraction is appropriate. In contrast, target sequences are generally longer, and using only a Transformer for global feature extraction was found to be less effective compared to MSCNN for capturing local features. Furthermore, a simple parallel approach did not yield significant improvements and performed slightly worse than MSCNN in parallel learning. However, when MSCNN and Transformer were used in parallel learning, followed by BFIM feature interaction and SFM feature selection and fusion, the overall performance improved significantly. This indicates that MCNN is highly effective in extracting local features of both drugs and targets, with the multi-scale nature ensuring a more comprehensive capture of these local features. For long target sequences, considering the secondary and tertiary structures as a whole, incorporating the Transformer allows for a more global and holistic extraction of the target’s structural information. The BFIM feature interaction and SFM feature fusion between MCNN and Transformer help to select effective global and local features, which further enhances the model’s predictive performance.

### 3.2. Model Comparison

The MCF-DTI model was compared with other representative methods, as shown in Table 2.

**Figure 1:**
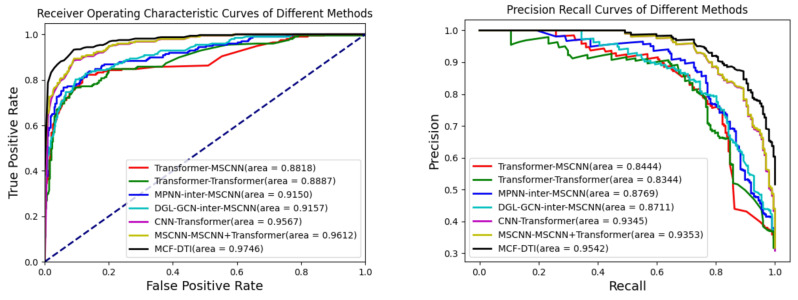
AUROC and AUPR scores of different methods.

The results presented in Table 2 and Figure 1 indicate that the MCF-DTI model outperforms all other compared models in every evaluated metric, including AUC, AUPR, Precision, Recall, and Accuracy. Specifically, MCF-DTI achieves the highest AUC of 0.9746, which reflects its superior ability to distinguish between interacting and non-interacting drug–target pairs. The AUPR of 0.9542 further demonstrates the model’s effectiveness in maintaining a high balance of precision and recall, particularly in identifying true positives among predicted positive samples. The recall value of 0.9036 highlights MCF-DTI’s strong capacity to identify most of the true interactions, while the precision value of 0.8696 indicates its accuracy in minimizing false positives. In comparison, models such as MSCNN-MSCNN+Transformer (C) and CNN-Transformer perform reasonably well, with AUCs of 0.9612 and 0.9567, respectively, but do not achieve the same level of overall performance. The Transformer-MSCNN and Transformer-Transformer models have relatively lower performance metrics, suggesting that incorporating multi-scale convolutional features, as performed in MCF-DTI, is crucial for effective feature extraction.

A review of recent publications on DTI prediction using the Davis dataset reveals that models such as MdDTI-ICMF (AUC 0.918) [30], MdDTI-ESPF (AUC 0.916) [30], HyperAttentionDTI (AUC 0.920) [35], and MolTrans (AUC 0.900) [36] all exhibit lower performance compared to our proposed model. This highlights the superiority and advanced capabilities of our approach in DTI prediction.

### 3.3. Case Validation and Prediction Studies

**Figure 2:**
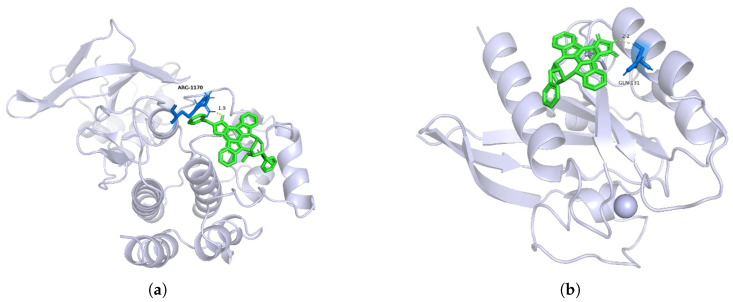
Molecular docking images for (**a**) target MET with compound CHEMBL415233 and (**b**) target KRAS with compound CHEMBL144479. The target proteins are represented in Ribbon mode (light blue), the ligands in Stick mode (green), and the binding pocket residues in Ball-and-Stick mode (blue). Key interactions such as hydrogen bonds (yellow dashed lines) and their distances (Å) are highlighted. (**a**) illustrates MET interacting with CHEMBL415233 via hydrogen bonds, while (**b**) depicts KRAS interacting with CHEMBL144479 through hydrophobic contacts.

To explore the reliability of the model’s predictions, we considered the KIBA dataset, where the number of biologically active compounds identified in drug or literature studies is over three times greater than that in the Davis dataset. Using lung cancer targeted therapy as an example [37], we predicted the likelihood of potential targeted therapies for the HER2, MET, and KRAS targets. We selected several approved drugs, six investigational targets, and the aforementioned HER2, MET, and KRAS targets along with their corresponding compounds for Autodock molecular docking experiments to obtain their binding energies. Subsequently, we analyzed the reliability of lung cancer target–potential drug pairs based on the binding affinities between drugs and targets. The binding energy results for nine target–drug pairs as predicted by the MCF-DTI model are shown in Table 3. Molecular docking images for target MET with the active compound CHEMBL415233 and target KRAS with active compound CHEMBL144479 are shown in Figure 2. The biologically active compounds predicted for HER2, MET, and KRAS are preclinical compounds, with Drug IDs corresponding to the CHEMBL dataset [38].

Autodock evaluates the binding affinity between a ligand and a receptor by calculating the minimum binding energy. Specifically, a binding energy lower than −4.25 kcal/mol indicates a certain degree of binding affinity. A binding energy lower than −5.00 kcal/mol suggests good binding between the ligand and receptor, while a binding energy lower than −7.00 kcal/mol indicates a strong binding affinity between the two molecules.

The analysis of Table 3 highlights the predictive probabilities and binding affinities for FDA-approved drugs, drugs under study, and those predicted by the MCF-DTI model. FDA-approved drugs, such as Gefitinib and Alectinib, along with drugs under investigation like Vemurafenib, exhibit high predictive probabilities (1.00) and favorable binding energies, indicating strong interactions. Predicted drugs for HER2, MET, and KRAS also show high probabilities (0.98–0.99) and strong binding affinities, supporting the reliability and effectiveness of the model’s predictions.

The FDA-approved drugs and investigational drugs along with their corresponding targets used in the case validation and prediction studies were primarily sourced from the DrugBank database. In contrast, the drugs predicted by the MCF-DTI model and their targets were mainly derived from the publicly available KIBA dataset. This design ensures the independence of the case validation and prediction studies while avoiding potential biases due to overlaps with the original training database.

Regarding the relationship between molecular docking and model predictions, molecular docking serves as an auxiliary analytical method. It provides biological rationalization for the model’s predictions but does not directly reflect the decision-making mechanism of the model.

Figure 2 displays the three-dimensional structure of the target in gray, with the small molecule ligands represented in green. The figure highlights the binding of the compounds to the active site of the target. In Figure 2a, the critical amino acid residue ARG-1170 (Arginine 1170) is labeled. This residue may interact with the ligand through hydrogen bonding, salt bridges, or hydrophobic interactions. The figure indicates an interaction distance of 1.9 Å, which is optimal for hydrogen bond formation, suggesting the presence of a hydrogen bond interaction. Based on the distance and the type of amino acid residue, it can be inferred that ARG-1170 might form a hydrogen bond with the ligand via its guanidinium group. This supports the hypothesis that an interaction exists between the target MET and the compound CHEMBL415233, indicating that CHEMBL415233 could serve as a targeted therapeutic agent for MET.

In Figure 2b, the key amino acid residue GLN-131 (Glutamine 131) and the ligand CHEMBL144479 are shown with an interaction distance of 2.2 Å. This suggests that GLN-131 may interact with the ligand through the amide group of its side chain, forming a hydrogen bond. Thus, it is confirmed that there is an interaction between CHEMBL144479 and the target KRAS.

## 4. Method

### 4.1. MCF-DTI Model Architecture

The primary challenge in DTI prediction lies in the complexity of drug and target features as well as the high-dimensional, nonlinear interactions between them. Designing an efficient model capable of automatically extracting features and deeply modeling drug–target interactions is of great significance. MSCNN and Transformer are powerful tools for extracting local and global features, respectively, making them well-suited for capturing the local features of drugs and the global features of targets. By combining these with a shared attention mechanism and sparse feature selection, the predictive performance of the model can be significantly enhanced.

**Figure 3:**
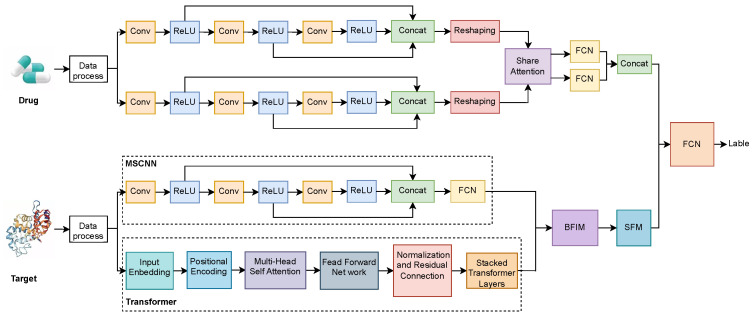
MCF-DTI Model Architecture.

As show in Figure 3, the proposed method aims to predict drug–target interactions by combining CNN and Transformer networks to extract and fuse multi-scale features from both drug and target data. The architecture incorporates a multi-path feature extraction structure that allows both drug and target information to be represented in diverse forms, followed by feature interaction and selection mechanisms for effective prediction.

On the drug side, the drug data is first pre-processed and then fed into two parallel multi-scale convolutional modules. Each of these multi-scale convolutional modules comprises multiple convolutional layers (Conv) followed by ReLU activation functions, extracting features from the drug at different levels and scales. This parallel multi-scale convolution structure helps to capture various features of the drug from different receptive fields, thus providing a richer feature representation. The features from each convolutional path are concatenated via a Concatenation operation, and subsequently reshaped to facilitate interaction with the features from the target side.

On the target side, after the initial data processing, the input flows into two parallel feature extraction paths. The first path employs an MSCNN, similar to the drug side, to extract the target features at different scales. The second path utilizes a Transformer network, which consists of input embedding, positional encoding, multi-head self-attention, feed-forward networks, and stacked Transformer layers. The multi-scale CNN pathway captures local features of the target, while the Transformer captures complex global relationships within the target sequence, resulting in a comprehensive feature representation.

The features extracted from both the drug and target sides are first processed through a shared attention mechanism to interactively fuse the information, obtaining the interrelated features between the drug and the target. These features are then passed through the Biased Feature Interaction Module (BFIM) to further enhance the interaction between drug and target features. Subsequently, the Sparse Feature Module (SFM) is employed to perform feature selection, retaining the most important features while eliminating redundancy. Finally, the concatenated features from the drug and target sides are combined through a Concatenation operation. The fused features are then passed to a Fully Connected Network (FCN) for prediction, providing the output interaction label for the drug–target pair.

This method effectively integrates features from both the drug and target sides, utilizing multi-level information interaction and filtering to ensure that the most critical features of drug–target interactions are extracted. In terms of data preparation, drugs are input as normalized SMILES strings, and targets are provided as FASTA-format protein sequences. The model supports input as drug–target pairs or as separate files. It is capable of correctly parsing the input and making predictions.

The core formula of the model is as follows:

1. Parallel Multi-Scale Convolution on Drug Side:
  (1) $$F_{\mathrm{drug},\;1}={\mathrm{Conv}}_{3}\left(\mathrm{ReLU}\left({\mathrm{Conv}}_{2}\left(\mathrm{ReLU}\left({\mathrm{Conv}}_{1}\left(X_{\mathrm{drug}}\right)\right)\right)\right)\right)$$
  (2) $$F_{\mathrm{drug},\;2}={\mathrm{Conv}}_{6}\left(\mathrm{ReLU}\left({\mathrm{Conv}}_{5}\left(\mathrm{ReLU}\left({\mathrm{Conv}}_{4}\left(X_{\mathrm{drug}}\right)\right)\right)\right)\right)$$
  The final drug feature is obtained by concatenating the outputs:
  (3) $$F_{\mathrm{drug}}=\mathrm{Concat}\left(F_{\mathrm{drug},\;1},F_{\mathrm{drug},\;2}\right)$$
2. Parallel Feature Extraction on Target Side:
  Multi-Scale Convolution Path:
  (4) $$F_{\mathrm{target},\;1}={\mathrm{Conv}}_{3}\left(\mathrm{ReLU}\left({\mathrm{Conv}}_{2}\left(\mathrm{ReLU}\left({\mathrm{Conv}}_{1}\left(X_{\mathrm{target}}\right)\right)\right)\right)\right)$$
  Transformer Path, including input embedding and multi-head self-attention:
  (5) $$\mathrm{Attention}\left(Q,K,V\right)=\mathrm{softmax}\left(\frac{QK^{T}}{\sqrt{d_{k}}}\right)V$$
  The final Transformer feature output:
  (6) $$F_{\mathrm{target},\;2}=\mathrm{Transformer}\left(X_{\mathrm{target}}\right)$$
  The final target feature is obtained by concatenation:
  (7) $$F_{\mathrm{target}}=\mathrm{Concat}\left(F_{\mathrm{target},\;1},F_{\mathrm{target},\;2}\right)$$
3. Feature Interaction and Selection:
  Feature cross through BFIM:
  (8) $$F_{\mathrm{cross}}=\mathrm{BFIM}\left(F_{\mathrm{drug}},F_{\mathrm{target}}\right)$$
  Feature selection through SFM:
  (9) $$F_{\mathrm{select}}=\mathrm{SFM}\left(F_{\mathrm{cross}}\right)$$
4. Final Prediction Output:
  (10) $$y=\mathrm{FCN}(\mathrm{Concat}\left(F_{\mathrm{drug}},F_{\mathrm{select}}\right))$$

### 4.2. Selective Fusion Module

**Figure 4:**
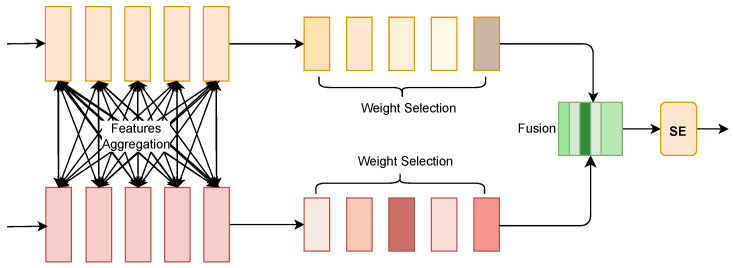
SFM Model Architecture.

As show in Figure 4, in the overall architecture, the Selective Fusion Module (SFM) is applied to the target side to process the features extracted from the MSCNN and Transformer. The purpose of SFM is to select the most representative features from these two different sources, namely, local and global features, thereby enhancing the efficiency and precision of drug–target interaction prediction.

First, the input data from the target side is processed through two parallel feature extraction paths: the convolution path, which captures local features, and the Transformer path, which captures global features. The features obtained from these two branches are denoted by *L* and *G*, respectively. These features are concatenated together to form a combined feature representation *X*:

(11) X=Concat(L,G)

Next, to make the computation more efficient, a dimensionality reduction is performed. The mean value of each row of the input tensor in the time dimension is computed, followed by an MLP layer with ReLU activation to reduce the feature dimension:

(12) $$I=F_{m}=\frac{1}{T}\sum_{i=1}^{T}x_{i},\;\hat{X}=F_{f}=\gamma \left(W_{f}I\right)$$

where $W_{f}$ represents the MLP weight matrix, and γ is the ReLU activation function. This step helps make the feature representation more compact and computationally efficient.

The reduced feature representation $\hat{X}$ is then processed by two separate MLP layers, $W_{u1}$ and $W_{u2}$, to restore it to the original dimension, producing two new feature representations $X_{1}$ and $X_{2}$:

(13) $$X_{1}=W_{u1}\left(\hat{X}\right),\;X_{2}=W_{u2}\left(\hat{X}\right)$$

These two features are concatenated again to form a new feature representation *Z*:

(14) $$Z=\mathrm{Concat}(X_{1},X_{2})$$

To determine which features are the most significant, the softmax function is applied to *Z*, producing a set of attention weights that are used to weight the original concatenated features *X*:

(15) $$X_{f}=\mathrm{Softmax}\left(Z\right)\times X$$

The softmax operation introduces a competition mechanism among the feature weights, helping to select the most appropriate features from both local and global aggregated features. Finally, the Squeeze-and-Excitation (SE) module is used to re-calibrate the output features, further enhancing the discriminative power of the selected features.

In summary, SFM on the target side effectively combines local features extracted by convolution and global features extracted by the Transformer. By selectively emphasizing the most relevant information, it provides a high-quality feature representation that contributes significantly to accurate drug–target interaction prediction.

## 5. Experiments

### 5.1. Datasets

The Davis [39] dataset is an essential benchmark for DTI prediction, widely used in bioinformatics and drug design research (see Table 4). In this study, we primarily utilized the Davis dataset. The original dataset consists of 30,056 drug–target binding affinity (Kd) records. Following standard practices, we classified drug–target pairs with Kd < 30 nM as positive samples, indicating strong binding affinity, while Kd ≥ 30 nM pairs were considered negative samples. A total of 1506 positive samples were identified, and to maintain data balance, we randomly selected 1506 negative samples. These balanced samples formed the foundation for subsequent model training and evaluation.

The constructed drug–target pairs in this study underwent a uniqueness check to ensure no duplicate entries were included. While the random selection process may potentially include false negatives (i.e., drug–target pairs with actual activity that were not experimentally identified as positive samples), this is a commonly adopted practice. In future studies, further analysis and evaluation of this issue can be conducted.

### 5.2. Data Preprocessing

In this study, the SMILES representation of drugs and the target protein sequences were preprocessed to make them suitable for training and prediction in deep learning models. The preprocessing pipeline involved multiple steps to convert these character-based sequences into numerical features.

1. Drug SMILES Representation Preprocessing
  First, the SMILES representations of the drugs were split into individual characters, resulting in a list of chemical symbols such as ‘C’, ‘O’, ‘=’, etc. After splitting, each character underwent a validation check to ensure that it was a legitimate SMILES character. Any unknown or invalid character was replaced with ‘?’. To maintain uniform input length, all SMILES sequences were either padded or truncated to a fixed maximum length, with padding characters represented by ‘?’ if the original sequence length was insufficient. This ensured that all SMILES representations had the same length. Finally, these characters were converted to numerical indices using a character-to-index mapping table and passed through an embedding layer, transforming them into high-dimensional vectors to capture the feature representation of each drug.
2. Target Protein Sequence Preprocessing
  For target protein sequences, each sequence was first split into individual amino acid characters, represented by their single-letter codes (e.g., ‘M’, ‘A’, ‘L’). These sequences were then validated to ensure that each character was one of the 20 standard amino acid symbols, with invalid characters replaced by ‘?’. To maintain consistency in the input data, all sequences were either padded or truncated to a fixed maximum length, ensuring that all protein sequences were of uniform length. For input to the Transformer, each protein sequence underwent Byte Pair Encoding (BPE), splitting the sequences into frequent subword units. These subunits were then mapped to numerical indices, and an input mask was generated to indicate the valid and padded positions within the sequence, allowing the Transformer to focus on meaningful input. After passing through the embedding layer, the target sequences were transformed into numerical vectors that allowed the model to further learn the features of the protein sequences.

These preprocessing steps ensured the uniformity and applicability of the drug SMILES and target protein sequence data, allowing them to be input into deep learning models in a standardized numerical format, enhancing the model’s effectiveness in predicting drug–target interactions.

### 5.3. Evaluation Metrics

Key metrics for evaluating the performance of a classification model include AUC, AUPR, Accuracy, Precision and Recall. Below is a detailed explanation of these metrics:

AUC (Area Under the ROC Curve): AUC is an important metric for evaluating the performance of a classification model, representing the area under the ROC curve. It assesses the model’s ability to distinguish between true positive and false positive rates at various thresholds. The higher the AUC, the better the model’s discriminative power.

AUPR (Area Under the Precision–Recall Curve): AUPR measures the area under the precision–recall curve. When AUPR approaches 1, it indicates that the model performs well in situations with class imbalance. AUPR is particularly useful in scenarios where the ratio of positive to negative samples is significantly skewed.

Accuracy: Accuracy refers to the proportion of correctly classified samples out of the total number of samples. It is a comprehensive metric that provides an overall measure of the model’s classification performance.

Precision: Precision reflects the proportion of true positive samples among those identified as positive by the model. A high precision indicates a low false positive rate, meaning the model is effective in minimizing incorrect positive predictions.

Recall: Recall describes the proportion of actual positive samples that are correctly identified by the model. A high recall indicates that the model successfully identifies most of the positive cases, thereby reducing the number of false negatives.

### 5.4. Parameter Settings

Throughout the experimental process, various hyperparameters of the model were continually adjusted, and the final parameters were set as follows: the learning rate was set to 0.001, the batch size was 64, and the model was trained for 500 epochs. On the drug side, three convolutional layers were configured with output channels of (32, 64, 96), and kernel sizes of (4, 6, 8). Similarly, on the target side, three convolutional layers were used with output channels of (32, 64, 96) and kernel sizes of (4, 8, 12). All other modules were configured using default settings. The model employed a 5-fold cross-validation strategy to ensure robust and reliable results. The experiments were conducted in an environment running Ubuntu 22.04.4, with 64 GB of memory, and the computations were accelerated using an NVIDIA GeForce RTX 3090 GPU.

### 5.5. Comparisons

To evaluate the robustness and reliability of the MCF-DTI model, several baseline models were constructed and compared on the Davis dataset. Each baseline model focuses on different feature extraction strategies, providing insights into their contributions to DTI prediction:

Transformer-MSCNN: This model uses a Transformer to extract global drug features and an MSCNN to capture both local and global features from the target protein.

Transformer-Transformer: Both the drug and target features are extracted using Transformers to assess the impact of a fully global feature extraction strategy on DTI prediction.

MPNN-inter-MSCNN: A Message Passing Neural Network (MPNN) [40] is used for extracting topological features from drugs, while an inter-MSCNN extracts multi-scale features from the target.

DGL-GCN-inter-MSCNN: This model employs a Graph Convolutional Network (GCN) [41] using the Deep Graph Library (DGL) [42] to extract graph-level drug features, while an inter-MSCNN extracts target features.

CNN-Transformer: A traditional CNN is used to extract local drug features, while a Transformer extracts global target features.

MSCNN-MSCNN+Transformer (C): This model uses an MSCNN for drug feature extraction and both MSCNN and Transformer for target feature extraction, providing a comprehensive set of local and global features.

Through these comparisons, we not only aim to demonstrate the superior performance of MCF-DTI under different feature extraction strategies but also to illustrate its robustness in DTI prediction, outperforming various state-of-the-art models in accuracy and consistency.

## 6. Conclusions

In this study, we proposed a novel approach to predict drug–target interactions (DTI) by constructing the MCF-DTI model, which utilizes raw features extracted from the SMILES representations of drugs and targets. These features were processed through multi-scale convolution to capture detailed local patterns of drugs, while multi-scale convolution combined with Transformer was used to achieve BIFM feature interaction and SFM feature fusion for comprehensive feature processing. The experimental results demonstrated that the MCF-DTI model achieved an AUC of 97.46% and an AUPR of 95.42%, indicating superior predictive performance. Furthermore, compared to a series of previously reported models, MCF-DTI showed a significant advantage, further validating the effectiveness of our proposed methodology.

We fully recognize the critical role of enantiomerism in drug activity and toxicity. Although the current model does not explicitly account for enantiomeric effects, future work will incorporate datasets containing enantiomer information and leverage three-dimensional molecular features and graph neural networks to model the specific impact of enantiomers. Regarding the “black-box” nature of the model, we aim to enhance its credibility and transparency by integrating experimental validation and advanced interpretability algorithms in future research. Additionally, we plan to develop an interactive visualization tool to allow real-time exploration of drug and target feature contributions, further supporting drug development. Finally, the model will be improved in terms of usability, supporting more flexible file formats, and extended to non-kinase targets through sequence similarity analysis and transfer learning, thereby enhancing its applicability and versatility.

## Figures and Tables

**Table 1:** Ablation experiment evaluation of the MCF-DTI model.

Model	AUC	AUPR	Pre	Rec	Acc
MSCNN-Transformer	0.9478	0.9134	**0.9026**	0.7056	0.8862
MSCNN-MSCNN	0.9675	0.9491	0.8968	0.868	**0.9164**
MSCNN2-MSCNN+Transformer (S)	0.9659	0.9217	0.8636	0.868	0.9148
MSCNN2-MSCNN2	0.9680	0.9359	0.8125	0.9239	0.9084
MCF-DTI	0.9746	0.9542	0.8696	0.9036	0.9148

**Table 2:** Comparison of MCF-DTI with other representative methods.

Model	AUC	AUPR	Pre	Rec	Acc
Transformer-MSCNN	0.8818	0.8443	0.8218	0.7259	0.8633
Transformer-Transformer	0.8887	0.8344	0.7989	0.7462	0.8601
MPNN-inter-MSCNN	0.9156	0.8769	0.8788	0.7310	0.8826
DGL-GCN-inter-MSCNN	0.9157	0.8711	0.8645	0.6802	0.8654
CNN-Transformer	0.9567	0.9369	0.8367	0.8367	0.9068
MSCNN-MSCNN+Transformer (C)	0.9612	0.9335	0.8376	0.8376	0.8971
MCF-DTI	0.9746	0.9542	0.8696	0.9036	0.9148

**Table 3:** Study of drug–target pairs and predictive research.

Type	Target	Drug	Predictive Probability	Binding Energy (kcal/mol)
Drugs FDA approved	EGFR	Gefitinib	1.00	−6.44
	ALK	Alectinib	1.00	−9.40
	ROS1	Crizotinib	1.00	−5.82
Drugs under study	RET1	Cabozantinib	1.00	−5.49
	NTRK1	Larotrectinib	1.00	−4.64
	BRAF	Vemurafenib	1.00	−5.27
Drugs prediction by MCF-DTI	HER2	CHEMBL95825	0.99	−4.47
	HER2	CHEMBL451964	0.99	−4.77
	MET	CHEMBL36432	0.99	−4.81
	MET	CHEMBL415233	0.98	−8.53
	KRAS	CHEMBL481491	0.99	−6.37
	KRAS	CHEMBL144479	0.98	−7.72

**Table 4:** Number of DTIs for the Davis dataset.

Dataset	Drug	Target	Interaction
Davis	68	442	30,056

## Data Availability

Data obtained from https://github.com/hkmztrk/DeepDTA/tree/master/data (accessed on 15 October 2023).
